# Ethanol Exposure Induces Microglia Activation and Neuroinflammation through TLR4 Activation and SENP6 Modulation in the Adolescent Rat Hippocampus

**DOI:** 10.1155/2019/1648736

**Published:** 2019-11-12

**Authors:** Qing Li, Dexiang Liu, Fang Pan, Cyrus S. H. Ho, Roger C. M. Ho

**Affiliations:** ^1^Department of Medical Psychology and Medical Ethics, School of Basic Medical Sciences, Shandong University, Jinan, Shandong, China; ^2^Department of Psychological Medicine, National University of Singapore, Singapore; ^3^Institute for Health Innovation and Technology (iHealthtech), National University of Singapore, Singapore

## Abstract

The ethanol-induced toll-like receptor 4 (TLR4) signal activation of microglia and neuroinflammation are observed in both adolescent and adult rat brains, but the regulatory mechanisms of some TLR4 signaling-related factors in this process are still unclear. SUMO-specific protease 6 (SENP6) inhibits neuroinflammation by dampening nuclear factor kappa-B (NF-*κ*B) activation via the de-SUMOylation of NF kappa-B essential modulator (NEMO). This study investigates the effects of long-term ethanol consumption on neuroinflammation in the hippocampus of adolescent rats and the regulatory roles of TLR4 and SENP6. Twenty-one days of ethanol exposure in adolescent rats were used to develop an animal model. The number of microglia, microglial activation, and the expression of TLR4 in the hippocampus of adolescent rats were examined by immunoreactivity. The levels of TLR4, activation of NF-*κ*B including IkB-*α* and p-NF-*κ*B-p65, and SENP6 were measured by western blotting. Proinflammatory cytokines including TNF-*α*, IL-1*β*, and IL-6 were measured by enzyme-linked immunosorbent assay. The NF-*κ*B activation and proinflammatory cytokines released in overexpressed SENP6 and siRNA targeting SENP6 microglial cells after treatment with ethanol were estimated in vitro. This study found that alcohol exposure increased the number of activated microglia and the levels of p-NF-*κ*B-p65 and proinflammatory cytokines, while it decreased the SENP6 level in wild-type rats, but not in TLR4 knockout rats. The ethanol-induced increases of p-NF-*κ*B-p65, TNF-*α*, and IL-1*β* were dampened by overxpression of SENP6 and enhanced in SENP6-siRNA microglia. Our data suggest that ethanol exposure during adolescence induces the microglia-mediated neuroinflammation via TLR4 activation, and SENP6 plays an essential role in dampening NF-*κ*B activation and neuroinflammation.

## 1. Introduction

The percentage of alcohol consumption in adolescents is increasing [1]. An adolescent brain is sensitive to alcohol than that of adults because the adolescent brain is undergoing higher levels of neuronal plasticity, synaptic remodeling, and neurogenesis [2–4]. Among the subregions of the brain, the adolescent hippocampus is particularly vulnerable to alcohol-induced structural damage which results in behavioral deficits [5, 6]. Studies showed that excessive alcohol consumption during adolescence reduces hippocampal volume and impairs hippocampal-dependent learning and memory [2, 7, 8]. More importantly, hippocampal neurogenesis, a process that continues throughout life and contributes significantly to structural and functional aspects of the hippocampus, is inhibited by alcohol exposure [9, 10].

Excessive ethanol consumption causes neurodegeneration [11, 12]. Neuroinflammation is hypothesized to be one pathogenetic process leading to alcoholic brain damage [13–15]. In the process of ethanol-induced neuroinflammation, activation of microglia plays a pivotal role particularly through toll-like receptor 4 (TLR4) activation [15–17]. TLR4 is an important member of pattern recognition receptors (PRR), which can be stimulated by pathogen-associated molecular patterns (PAMPs) and danger-associated molecular patterns (DAMPs) and then trigger microglia release of proinflammatory cytokines [18–20]. Previous studies have observed increased TLR4 level and endogenous TLR4 activators such as high mobility group box-1 protein (HMGB1) in mouse brains following chronic and excessive ethanol exposure and in postmortem brains from alcoholics [21–23]. In contrast, knockdown of TLR4 or blockade of TLR4 activation by selective inhibitors prevents alcohol-induced increase of Iba-1 immunoreactivity and proinflammatory cytokines [24–27]. These findings suggest the critical role of TLR4 in microglia-mediated neuroinflammation following alcohol exposure.

Nuclear factor kappa-B (NF-*κ*B), a pivotal nuclear transcription factor in the TLR4 signal pathway, participates in both the MyD88- and TRIF-dependent pathways and plays an important role in the transcription and synthesis of inflammatory factors [28]. The activation of NF-*κ*B depends on the activity of the inhibitor of NF-*κ*B (IkB) kinase (IKK) complex, which makes up an essential regulatory subunit—the NF-*κ*B essential modifier (NEMO/IKKc)—required for NF-*κ*B activation. Intriguingly, NEMO is modified by the polyubiquitin chain, which is also critical for IKK activation [29, 30]. The deubiquitinase cylindromatosis (CYLD) could interact with NEMO and cleave these polyubiquitin chains, thus acting as a negative regulator of NF-*κ*B activation [31]. Specifically, the small ubiquitin-like modifier 2/3 (SUMO-2/3) is conjugated onto the lysine residue 277 of NEMO, impairing the deubiquitinase CYLD from binding with the NEMO and thus strengthening the IKK activation. SUMO-specific protease 6 (SENP6) can specifically reverse this process by catalyzing the de-SUMOylation of NEMO [32]. The overexpression of SENP6 significantly decreased the lipopolysaccharide- (LPS-) induced release of proinflammatory proteins, while the depletion of SENP6 significantly increased these proteins, indicating that SENP6 could regulate inflammation via dampening NF-*κ*B activation [33].

Based on the aforementioned studies, the present study is aimed at assessing the microglial activation, TLR4 expression, proinflammatory cytokine release in the hippocampus of adolescent rats after chronic excessive ethanol exposure, the roles of TLR4 and SENP6 in ethanol-induced neuroinflammation.

## 2. Experimental Method

### 2.1. Animals and Treatment

Twenty adolescent male Wistar rats (WT), with a body weight of 55-70 g and an age of 21-23 days, were obtained from the Animal Experimental Center of Shandong University. Twenty TLR4 knockout rats (KO) from Wistar background with a body weight of 53-71 g and an age of 21 days were produced by transcriptional activator-like effector nuclease- (TALEN-) mediated gene inactivation and sponsored by Dr. Chen's Laboratory of Shandong University (Jinan, Shandong, China) [34]. All rats were allowed to acclimate for one week after arrival. The rats were housed in groups (4-6 rats/cage) and maintained at a constant temperature (22 ± 1.0°C) and humidity (55 ± 5%), on an artificial 12 h light/dark cycle with lights on at 06:00 am. Purina Rodent Chow and tap water were available ad libitum, and the rats were weighed every 3 days. All procedures were in accordance with the principles of laboratory animal care of the China Laws for the Protection of Animals and approved by the Ethics Committee of Shandong University.

After the 7-day acclimatization period, the 20 WT rats and 20 KO rats were randomly assigned to four groups: WT control (WT) group (*n* = 10), ethanol-treated WT (WT+EtOH) group (*n* = 10), KO control (KO) group (*n* = 10), and ethanol-treated KO (KO+EtOH) group (*n* = 10). Rats in the WT+EtOH and KO+EtOH groups had free access to a 10% aqueous ethanol (*v*/*v*), whereas rats in the WT and KO groups received tap water. After 21 days of treatment, all rats were sacrificed.

The rats were exposed to Purina Rodent Chow ad libitum during the experiment. The ethanol bottles were weighed at 20:00 each day to determine the ethanol consumption of each sample. Then, the ethanol solution was refreshed by the dilution of anhydrous alcohol (Gold Shield Chemicals, Hayward, CA) with tap water and weighed again. The ethanol consumptions (g/kg body weight/24 h) were calculated for each rat, and values were averaged across 21 days of treatment.

### 2.2. Culture of Microglial Cells

Primary rat cultures of mixed glial cells were prepared, and the microglial cells were purified as previously described [35] with some modification. In aseptic conditions, the brains of 1-day-old neonatal rats were removed and carefully stripped off the meninges and blood vessels. After washing with sugared D-Hanks, the tissues were cut into 2 mm × 2 mm × 2 mm and digested with 0.125% trypsin for 15 min at 37°C. The supernatants were collected, centrifuged with 1000 r/min for 10 min, and resuspended in the culture medium after removing the supernatants. Cells were seeded in a 75 cm culture flask at a density of 250,000 cells/mL and maintained at 37°C in a humidified environment containing 5% CO_2_. The medium was replaced every 4 days. Ten days later after shaking at 260 r/min for 2 h at 37°C, the culture media were collected and seeded in new flasks for 1 h. The anchorage-dependent cells, known as microglia, were obtained. Then, the cell culture was stained by immunofluorescence using anti-Iba-1 and DAPI for purity identification. The nuclei in the DAPI staining appeared blue, and Iba-1-positive (Iba-1^+^) cells appeared red. The merging of blue and red was considered positive cells (microglia) when counting. All counting was performed by researchers who were blinded to the groups. Cells in seven random fields (200x magnification) were quantified and averaged. The microglial purity (%) = number of positive cells/total number of nuclei × 100%. Our data showed that the number of positive cells was 304.43 ± 3.56 cells/field, the total number of nuclei was 319.43 ± 3.43 cells/field, and the microglia purity was 95.30 ± 0.27%. The microglial cells were then treated with ethanol at different doses (10, 30, 50, and 100 mM) for 24 h, and the SENP6 protein levels were measured by the WB to detect the relation of ethanol and SENP6 protein expression. The ethanol concentrations used in the present study were in the range of the blood alcohol levels found among alcoholics (10-120 mM) [36].

### 2.3. Blood Ethanol Levels

Tail blood samples were collected weekly at two time points, namely, 2 h after the onset of light and dark. Briefly, the blood was centrifuged at 2000 r/min for 5 min. Serum was collected and stored at –80°C until further analysis. The blood ethanol concentrations (BECs) (mg/dL) from the serum were determined with a commercial colorimetric assay (MAK076, Sigma). Briefly, 10 *μ*L of each sample was added into a 96-well plate and diluted in Ethanol Assay Buffer to a final volume of 50 *μ*L. Then, after adding 50 *μ*L of the Master Reaction Mix to each of the wells, the solution was mixed well using a horizontal shaker and incubated for 30 minutes at 37°C, and the absorbance was measured at 570 nm. All samples and standards were made in duplicate. The blood ethanol concentrations obtained from the three time points were averaged for each animal.

### 2.4. Tissue Preparation

At the end of the treatment, 4 rats from each group (*n* = 4) were sacrificed for fluorescent immunohistochemical staining. Briefly, the animals were anesthetized with an intraperitoneal injection of sodium pentobarbital (80 mg/kg body weight) and perfused with saline intracardially until the liver turned pale pink, followed by 4% buffered formalin. Then, the brains were removed and fixed in formalin for 24 h. The brain samples were dehydrated and embedded in paraffin wax. Serial sections of the hippocampus were cut at 5 *μ*m intervals. The remaining 6 rats of each group (*n* = 6) were sacrificed for WB or ELISA analysis. After decapitation, the brains were rapidly removed from the skulls, and the hippocampal formations were dissected, quickly frozen in liquid nitrogen, and stored at –80°C until use.

### 2.5. Fluorescent Immunohistochemical Staining and Micrograph Analysis

Both quiescent and activated microglia were labeled with the Iba-1 antibody, the phagocytic activated microglia were labeled with ED-1 antibody, and the expressive level of TLR4 was labeled with the TLR4 antibody. After being deparaffinized with xylene, rehydrated with a descending alcohol series, and washed with distilled water, the sections were placed in a repair box full of EDTA antigen repair buffer (pH 8.0) for 15 min antigen retrieval in a microwave. Then, after washing in 0.01 M phosphate-buffered saline (PBS) (pH 7.4) for three bouts of 5 min, spontaneous fluorescence quencher was added to the sections for 5 min, which were then blocked for 30 min in normal goat serum (5% in PBS) in a humid chamber at 37°C and then incubated with polyclonal rabbit anti-Iba-1 primary antibody (1 : 500) (GB1305-1, Wuhan Service Biotechnology Co.), polyclonal rabbit anti-ED-1 primary antibody (1 : 100) (GB11067, Wuhan Service Biotechnology Co.), and polyclonal mouse anti-TLR4 primary antibody (1 : 200) (GB12186, Wuhan Service Biotechnology Co.) at 4°C overnight. After being diluted three times in PBS containing 0.03% Triton X-100 for 5 min, sections were incubated with the CY3 goat anti-rabbit secondary antibody (1 : 300) (GB21303, Wuhan Service Biotechnology Co.) for Iba-1 and ED-1 staining or FITC goat anti-mouse secondary antibody (1 : 400) (GB25301, Wuhan Service Biotechnology Co.) for TLR4 staining for 50 min at 37°C and added with 2-(4-amidinophenyl)-6-indolecarbamidine dihydrochloride (DAPI) staining solution for 5 min at 37°C after washing in PBS (pH 7.4) for three bouts of 5 min. Finally, the sections were mounted with mounting medium containing antiquenching fluorescence and observed using an inverted fluorescence microscope (Nikon Eclipse TI-SR, Nikon, Japan). Image capturing was performed using an imaging system (Nikon DA-U3, Nikon, Japan). The nuclei in the DAPI staining appeared blue, Iba-1-positive (Iba-1^+^) or ED-1-positive (ED-1^+^) cells appeared red, and TLR4-positive (TLR4^+^) receptors appeared red when simple staining or green when double staining with ED-1. The merging of blue and red (Iba-1^+^, ED-1^+^, or TLR4^+^ cells) and the merging of blue, red, and green (ED-1^+^/TLR4^+^ colabeling cells) were considered when counting. All counting was performed by researchers who were blinded to the groups. Cells in five random fields (400x magnification) across each region of interest (CA1, CA2/3, and DG) in three consecutive sections of each rat were quantified. The numbers of cells obtained from the three sections were averaged for each animal, and four animals were used for each fluorescent immunohistochemical staining. The results were expressed as the number of cells per mm^2^.

### 2.6. Western Blot Analysis

The WB assay was modified as previously reported [37]. Brain tissue samples (*n* = 6 per group) and cultured cells were homogenized in lysis buffer. The homogenate was centrifuged at 14,000 × *g* for 30 min at 4°C. The supernatant was stored at −80°C until use. The protein concentration was measured using a Protein Quantitative Analysis Kit (k3001-BCA; Shenergy Biocolor, Shanghai, China) from the Bio-Rad DC. Equal amounts of protein (50 *μ*g) from each sample were boiled in 6×Laemmli loading buffer for 5 min, run on 12.5% (for IkB-*α* and p-NF-*κ*B-p65) or 8% (for TLR4 and SENP6) SDS-polyacrylamide gels, and then transferred to 0.2 *μ*m polyvinylidene fluoride (PVDF) membranes (Millipore Corporation). The blots were blocked for 2 h in blocking solution (5% nonfat dry milk, 0.05% Tween-20 in PBS) at 37°C, then incubated overnight with anti-sera (dilution): *β*-actin (1 : 2000) (#4967, Cell Signaling Corp., Beverly, CA, USA), SENP6 (1 : 1000) (ab77619, Abcam), IkB-*α* (1 : 1000) (ab32518, Abcam), p-NF-*κ*B-p65 (1 : 1000) (ab183559, Abcam), TLR4 (1 : 1000) (sc-293072, Santa Cruz Biotechnology), GAPDH (1 : 1000) (ab8245, Abcam), Lamin A/C (1 : 500) (ab8984, Abcam) at 4°C, and visualized using horseradish peroxidase- (HRP-) conjugated goat anti-rabbit (A0208, Beyotime Institute of Biotechnology, Shanghai, China), donkey anti-goat (A0180, Beyotime Institute of Biotechnology, Shanghai, China), goat anti-mouse (ab205719, Abcam) secondary antibodies (1 : 20000), and an ECL detection system (Millipore Corporation). The bands corresponding to *β*-actin, SENP6, IkB-*α*, p-NF-*κ*B-p65, TLR4, GAPDH, and Lamin A/C were scanned and densitometrically analyzed using ImageJ software. These quantitative analyses were normalized to *β*-actin/GAPDH/Lamin A/C (after stripping).

### 2.7. ELISA Analysis

The samples of brain tissues and cultured cells were prepared as WB. The protein levels of TNF-*α* (#KRC3011, Invitrogen), IL-1*β* (ab100768, Abcam), and IL-6 (#BMS625, Invitrogen) were determined by ELISA according to the manufacturer's instructions. The standards were run in duplicate and samples in triplicate. Absorbance was measured at 450 nm using the Bio-Rad DC. Cytokine concentrations were normalized to the total protein content and reported as pg/mg of total protein ± S.E.M.

### 2.8. Construction of the Overexpressed SENP6 or siRNA Targeting SENP6 Plasmid and Transfection

SENP6-modified plasmids were prepared using routine methods. Briefly, the constructs for overexpressed SENP6 (SENP6), siRNA targeting SENP6 (siSENP6), and control scrambled sequence (Scr) were cloned into pcDNA3.1–EGFP to generate the corresponding plasmids. The sequences were as follows: SENP6: 5′-AACUGCAACUUCUAAACACAAAA-3′, siRNA SENP6: 5′-GGGUGAUAAAGCCUGUAAATT-3′, and Scr: 5′-UUCUCCGAACGUGUCACGUTT-3′. These plasmids of 2 *μ*g were transfected into cultured rat microglia using Lipofectamine 2000, according to the manufacturer's instructions (Invitrogen). The plasmids also contained a GFP reporter to allow the determination of the transfection efficiency, which was nearly 100% in the current study. Then, we measured the SENP6 protein level to confirm the transfection effect 48 h later. Half of the Scr group (Scr-E) and the siSENP6 and SENP6 groups were treated with 100 mM ethanol for 24 h, and the remaining Scr group with no treatment as nonethanol control group (Scr-Con). Twenty-four hours later, we measured the SENP6, IkB-*α*, and p-NF-*κ*B-p65 protein levels by WB, and the TNF-*α*, IL-1*β*, and IL-6 protein levels by ELISA to assess the effect of ethanol on activation of NF-*κ*B and associated neuroinflammation in microglial cells and the modulatory role of SENP6 in this process.

### 2.9. Statistical Analysis

Data were presented as means and standard errors. Data from three or more groups were analyzed using one-way analysis of variance (ANOVA) with treatment as the independent variable. Post hoc analyses were performed whenever appropriate, using the Turkey test when normality assumptions were satisfied. Comparison between two groups was made with an independent-sample *t*-test. A result of *p* < 0.05 was considered to be statistically significant. All statistical analyses were conducted with the SPSS17.0 software.

## 3. Results

### 3.1. Blood Ethanol Concentrations

During the 21 days of ethanol treatment, the adolescent rats consumed 9.74 ± 0.67 g of ethanol per kilogram of body weight daily in the WT+EtOH group and 9.57 ± 0.71 g of ethanol per kilogram of body weight daily in the KO+EtOH group. There were no significant differences between the WT+EtOH and KO+EtOH groups in the ethanol consumption (*t* (18) = 0.167, *p* = 0.869). The blood ethanol concentrations were 34 ± 3.2 mg/dL 2 h after light onset and 105 ± 7.1 mg/dL 2 h after light offset in the WT+EtOH group and 32 ± 2.7 mg/dL 2 h after light onset and 98 ± 7.2 mg/dL 2 h after light offset in the KO+EtOH group. There were no significant differences across the three time points (1st week, 2nd week, and 3rd week) both at 2 h after light onset (*F* (1, 18) = 0.511, *p* = 0.484) and at 2 h after light offset (*F* (1, 18) = 0.836, *p* = 0.373) in the WT+EtOH group, as well as in the KO+EtOH group (2 h after light onset: *F* (1, 18) = 0.078, *p* = 0.783; 2 h after light offset: *F* (1, 18) = 0.100, *p* = 0.756). There were no significant differences between the WT+EtOH and KO+EtOH groups across the three time points of both 2 h after light onset (1st week: *t* (18) = 0.131, *p* = 0.897; 2nd week: *t* (18) = 0.346, *p* = 0.734; 3rd week: *t* (18) = 0.059, *p* = 0.954) and 2 h after light offset (1st week: *t* (18) = 0.096, *p* = 0.924; 2nd week: *t* (18) = 0.698, *p* = 0.494; 3rd week: *t* (18) = 0.955, *p* = 0.352).

### 3.2. The Iba-1 Expression in Immunoreactivity

**Figure 1:**
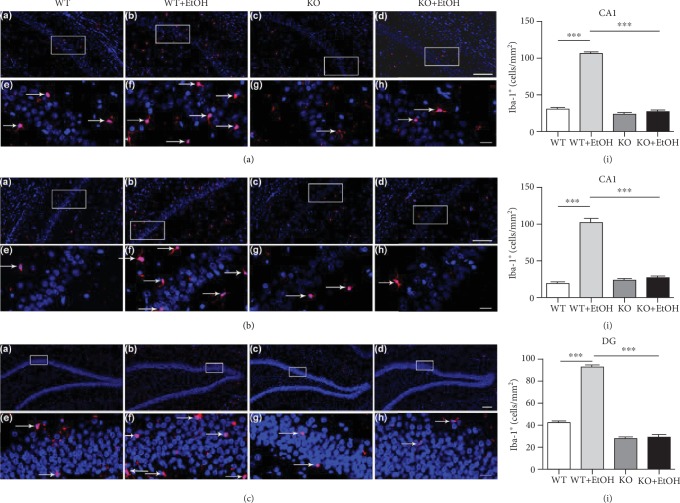
Effect of ethanol exposure on the number of microglia (Iba-1 staining) in the hippocampus. Representative photomicrographs of level-matched coronal sections of CA1 (a, A–H), CA2/3 (b, A–H), and DG (c, A–H) from WT (A, E), WT+EtOH (B, F), KO (C, G), and KO+EtOH (D, H) rats. White arrows indicate Iba-1^+^ microglia. Regions of interest (framed areas) in A–D are presented in E–H. Scale bar: 100 *μ*m in A–D; 20 *μ*m in E–H. WT = control rats without ethanol; WT+EtOH = control rats with ethanol; KO = TLR4 knockout rats without ethanol; KO+EtOH = TLR4 knockout rats with ethanol. CA1 = field CA1 of the hippocampus; CA2/3 = field CA2-3 of the hippocampus; DG = dentate gyrus of the hippocampus. Quantifications of Iba-1^+^ microglia from WT, WT+EtOH, KO, and KO+EtOH rats are shown in (a, I) (CA1), (b, I) (CA2/3), and (c, I) (DG). The number of Iba-1^+^ microglia was averaged from five random areas across each region in consecutive three sections of each rat. Data are presented as the mean number of Iba-1^+^cells/mm^2^ ± S.E.M. (*n* = 4). ^∗∗^*p* < 0.01, ^∗∗∗^*p* < 0.001.

As shown in Figures 1(a)–1(c), the numbers of Iba-1^+^ microglia in the CA1 (*t* (6) = 25.354, *p* < 0.001), CA2/3 (*t* (6) = 13.594, *p* < 0.01), and DG (*t* (6) = 19.424, *p* < 0.001) of the hippocampus in the WT+EtOH group significantly increased after excessive ethanol exposure compared with the control groups. However, the increased Iba-1^+^ microglia in the three subfields of the hippocampus were noticeably reduced in the corresponding groups with TLR4 gene knockdown (CA1: *t* (6) = 22.669, *p* < 0.001; CA2/3: *t* (6) = 12.474, *p* < 0.001; DG: *t* (6) = 25.811, *p* < 0.001), and the levels of Iba-1 immunoreactivity were similar in both ethanol-treated and nontreated TLR4-KO rats (CA1: *t* (6) = 1.667, *p* = 0.147; CA2/3: *t* (6) = 0.585, *p* = 0.580; DG: *t* (6) = 0.067, *p* = 0.949). The results indicate that ethanol exposure increases Iba-1 immunoreactivity of microglia in the adolescent rat hippocampus depending on TLR4 activation.

### 3.3. The ED-1 and TLR4 Expression in Immunoreactivity

**Figure 2:**
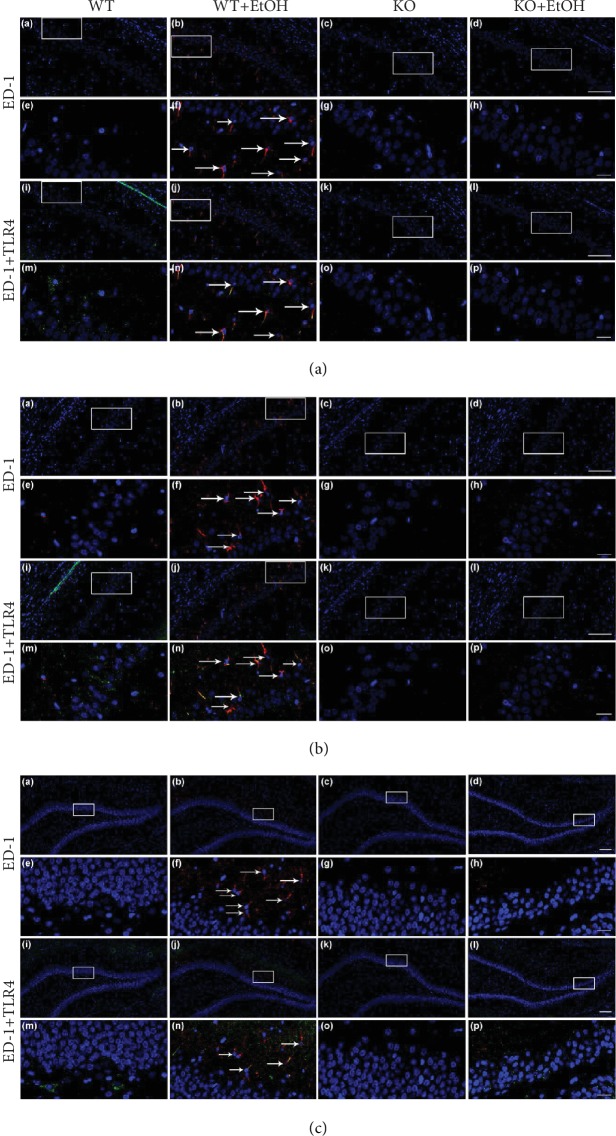
Effect of ethanol exposure on the extent of microglial activation (ED-1 staining) and colocalization of ED-1 with TLR4 (ED-1/TLR4 colabeling) in the hippocampus. Representative photomicrographs of level-matched coronal sections of CA1 (a, A–P), CA2/3 (b, A–P), and DG (c, A–P) from WT (A, E, I, M), WT+EtOH (B, F, J, N), KO (C, J, K, O), and KO+EtOH (D, H, L, P) rats. White arrows indicate ED-1^+^ (F) or ED-1^+^/TLR4^+^ (N) cells. Regions of interest (framed areas) in A–D are presented in E–H for ED-1 staining and in I–L are presented in M–P for ED-1/TLR4 colabeling. Scale bar: 100 *μ*m in A–D, I–L); 20 *μ*m in E–H, M–P. WT = control rats without ethanol; WT+EtOH = control rats with ethanol; KO = TLR4 knockout rats without ethanol; KO+EtOH = TLR4 knockout rats with ethanol. CA1 = field CA1 of the hippocampus; CA2/3 = field CA2-3 of the hippocampus; DG = dentate gyrus of the hippocampus.

As shown in Figures 2(a)–2(c), no ED-1^+^ cells were observed in any subfields of the hippocampus in the WT or TLR4-KO animals without ethanol treatment. However, chronic ethanol treatment markedly increased the number of ED-1^+^ cells in the WT rats, but not in TLR4-KO animals. The numbers of ED-1^+^ cells in the WT+EtOH group were 90.00 ± 8.42 and 90.67 ± 7.37 in the CA1 and CA2/3 regions and 95.11 ± 6.92 in the DG region. Furthermore, a large proportion of ED-1^+^ cells that highly expressed TLR4 was observed in the WT ethanol group; briefly, 80.2% and 70.3% of the ED-1^+^ cells colocalized with TLR4 in the CA1 and CA2/3 regions, and 53.7% of the ED-1^+^ cells were TLR4^+^ cells in the DG region in the WT+EtOH group. The data suggest that alcohol exposure drives microglia to a state of phagocytic activation and supports the key role of TLR4 in regulating ethanol-induced microglial activation.

### 3.4. The TLR4 Receptor Expression

**Figure 3:**
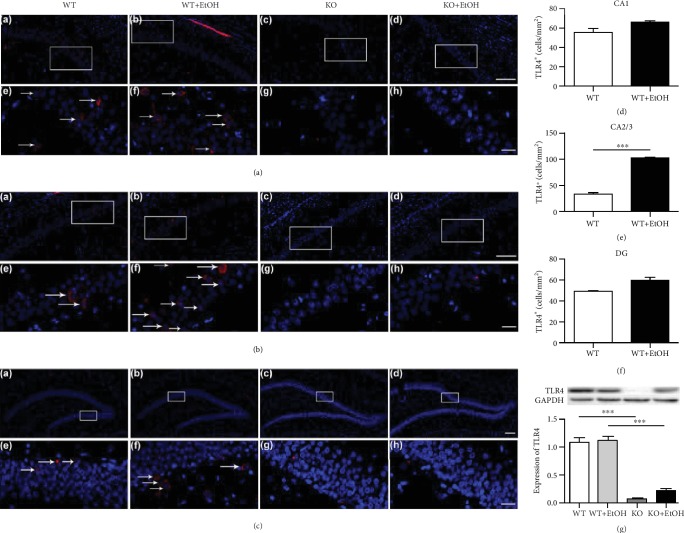
Effect of ethanol exposure on the TLR4 expression in the hippocampus. Representative photomicrographs of level-matched coronal sections of CA1 (a, A–H), CA2/3 (b, A–H), and DG (c, A–H) from WT (A, E), WT+EtOH (B, F), KO (C, G), and KO+EtOH (D, H) rats. White arrows indicate TLR4^+^ cells. Regions of interest (framed areas) in A–D are presented in E–H. Scale bar: 100 *μ*m in A–D; 20 *μ*m in E–H. WT = control rats without ethanol; WT+EtOH = control rats with ethanol; KO = TLR4 knockout rats without ethanol; KO+EtOH = TLR4 knockout rats with ethanol. CA1 = field CA1 of the hippocampus; CA2/3 = field CA2-3 of the hippocampus; DG = dentate gyrus of the hippocampus. Quantifications of TLR4^+^ cells from WT and WT+EtOH rats are shown in (d) (CA1), (e) (CA2/3), and (f) (DG). The number of TLR4^+^ cells was averaged from five random areas across each region in consecutive three sections of each rat. Data are presented as the mean number of Iba-1^+^cells/mm^2^ ± S.E.M. (*n* = 4). ^∗∗∗^*p* < 0.001. The hippocampal protein level of TLR4 (g) was measured by WB, and quantitative analyses were normalized to GADPH. Data are presented as the mean ± S.E.M. ^∗∗∗^*p* < 0.001.

Figures 3(a)–3(f) show the quantification of the TLR4^+^ cells in immunoreactivity which demonstrated that TLR4 expression only markedly increased in the CA2/3 region (*t* (6) = 12.810, *p* < 0.001) of the WT ethanol-treated animals but not in the CA1 (*t* (6) = 2.420, *p* = 0.052) or DG (*t* (6) = 1.895, *p* = 0.107) subfields. No TLR4^+^ cells were observed within the hippocampus of TLR4-KO rats. However, the quantification analysis of TLR4 protein level in WB showed no change in the WT groups with or without ethanol treatment (*t* (10) = 0.226, *p* = 0.826) (Figure 3(g)), suggesting that chronic ethanol exposure has no effect on TLR4 expression. Knockout of TLR4 significantly downregulated the TLR4 levels with or without ethanol treatment (WT vs. KO: *t* (10) = 8.942, *p* < 0.001; WT+EtOH vs. KO+EtOH: *t* (10) = 7.949, *p* < 0.001).

### 3.5. SENP6, IkB-*α*, p-NF-*κ*B-p65, and Inflammatory Cytokine Levels

**Figure 4:**
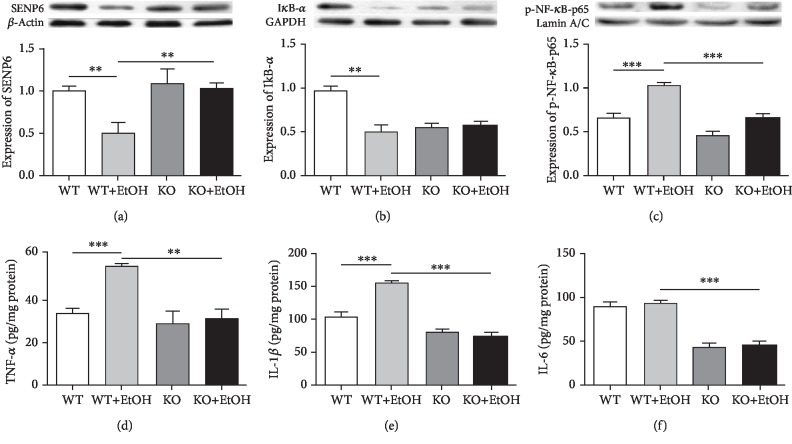
Effects of ethanol exposure on the levels of SENP6, IkB-*α*, p-NF-*κ*B-p65, and proinflammatory cytokines in the hippocampus. The hippocampal levels of SENP6 (a), IkB-*α* (b), and p-NF-*κ*B-p65 (c) of the WT, WT+EtOH, KO, and KO+EtOH rats were measured by WB. And the levels of TNF-*α* (d), IL-1*β* (e), and IL-6 (f) proteins were measured by ELISA. WT = control rats without ethanol; WT+EtOH = control rats with ethanol; KO = TLR4 knockout rats without ethanol; KO+EtOH = TLR4 knockout rats with ethanol. Quantitative analyses were normalized to *β*-actin (for SENP6), GADPH (for IkB-*α*), or Lamin A/C (for p-NF-*κ*B-p65) for WB. Cytokine concentrations were normalized to the total protein content. Data are presented as the mean ± S.E.M. ^∗∗^*p* < 0.01, ^∗∗∗^*p* < 0.001.

Figures 4(a)–4(f) show that the WT+EtOH group had decreased SENP6 (*t* (10) = 3.465, *p* < 0.01) and IkB-*α* levels (*t* (10) = 5.004, *p* < 0.001), increased levels of p-NF-*κ*B-p65 (*t* (10) = 7.346, *p* < 0.001), and increased levels of TNF-*α* (*t* (10) = 10.293, *p* < 0.001) and IL-1*β* (*t* (10) = 7.058, *p* < 0.001) compared with the WT group, while no significant increased IL-6 level was observed in the WT+EtOH group compared with the WT group (*t* (10) = 0.285, *p* = 0.789). Conversely, in TLR4-KO rats, no changes were seen in any index after ethanol treatment (SENP6: *t* (10) = 0.424, *p* = 0.680; IkB-*α*: *t* (10) = 0.288, *p* = 0.780; p-NF-*κ*B-p65: *t* (10) = 2.143, *p* = 0.057; TNF-*α*: *t* (10) = 0.209, *p* = 0.839; IL-1*β*: *t* (10) = 0.959, *p* = 0.360; IL-6: *t* (10) = 0.453, *p* = 0.660). Furthermore, the KO+EtOH group displayed significantly increased SENP6 level (*t* (10) = 3.365, *p* < 0.01), decreased p-NF-*κ*B-p65 level (*t* (10) = 6.149, *p* < 0.001), and lower levels of TNF-*α* (*t* (10) = 4.808, *p* < 0.01), IL-1*β* (*t* (10) = 14.915, *p* < 0.001), and IL-6 (*t* (10) = 6.437, *p* < 0.001) compared with the WT+EtOH group. The results indicated that ethanol exposure decreased SENP6 expression and activated NF-*κ*B inflammatory signaling via TLR4 activation.

### 3.6. Role of SENP6 in NF-*κ*B Activation and Proinflammatory Cytokine Release

**Figure 5:**
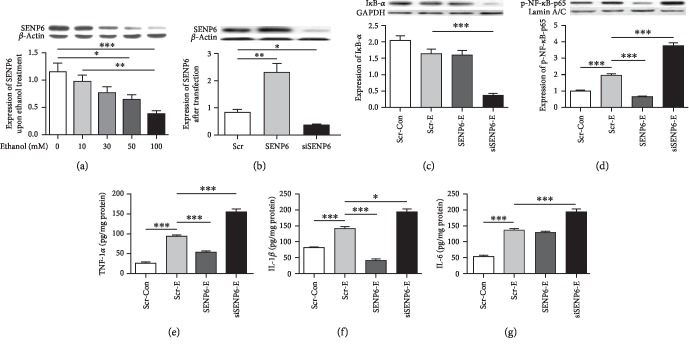
The modulatory effect of SENP6 in ethanol-induced neuroinflammation. (a) The statistical analyses of SENP6 levels in cultured microglial cells treated with different doses of ethanol (0, 10, 30, 50, and 100 mM) for 24 h. (b) The transfection efficiency by comparing the SENP6 level of the SENP6 and siSENP6 groups to Scr group. (c–g) The levels of IkB-*α*, p-NF-*κ*B-p65, TNF-*α*, IL-1*β*, and IL-6 in microglial cells of the Scr group with nonethanol treatment and of Scr, SENP6, and siSENP6 groups after treatment with ethanol at100mM for 24 h. Scr-Con = scrambled sequence group with nonethanol treatment; Scr-E = scrambled sequence group with 100 mM ethanol treatment; SENP6-E = overexpressed SENP6 group with 100 mM ethanol treatment; siSENP6-E = siRNA against SENP6 group with 100 mM ethanol treatment. Quantitative analyses were normalized to *β*-actin (for SENP6), GADPH (for IkB-*α*), or Lamin A/C (for p-NF-*κ*B-p65) for WB. And cytokine concentrations were normalized to the total protein content. Data are presented as the mean ± S.E.M. ^∗^*p* < 0.05, ^∗∗^*p* < 0.01, ^∗∗∗^*p* < 0.001.

Figure 5(a) shows the results of the SENP6 level in cultured microglial cells treated with different doses of ethanol (0, 10, 30, 50, and 100 mM) for 24 h. Ethanol dose dependently decreased the SENP6 levels (*F* (4, 25) = 7.246, *p* = 0.001) in cultured microglial cells with statistical significance at the dose of 50 mM (*p* < 0.05) and100 mM (*p* < 0.001), compared with the nonalcoholic control group.

Figure 5(b) shows that the modification of the SENP6 level in these cells which were first confirmed by western blot (Scr vs. SENP6: *t* (10) = 4.579, *p* < 0.01; Scr vs. siSENP6: *t* (10) = 3.618, *p* < 0.05).

Figures 5(c) and 5(d) show the IkB-*α* and p-NF-*κ*B-p65 levels. The level of p-NF-*κ*B-p65 (*t* (10) = 12.328, *p* < 0.001) was markedly increased versus controls when the cells were subjected to 100 mM ethanol. The overexpression of SENP6 significantly dampened the increased effects of ethanol on the level of p-NF-*κ*B-p65 (*t* (10) = 18.458, *p* < 0.001). The ethanol-induced activations of the p-NF-*κ*B-p65 (*t* (10) = 7.003, *p* < 0.001) was enhanced in the SENP6-siRNA-transfected cells.

Figures 5(e)–5(g) shows the levels of TNF-*α*, IL-1*β*, and IL-6 in normal, SENP6, and siSENP6-transfected microglia after treatment with 100 mM ethanol. The levels of TNF-*α* (*t* (10) = 8.989, *p* < 0.001), IL-1*β* (*t* (10) = 5.540, *p* < 0.001), and IL-6 (*t* (10) = 10.592, *p* < 0.001) were markedly increased versus controls when the cells were subjected to 100 mM ethanol. The overexpression of SENP6 significantly dampened the increased effects of ethanol on the levels of TNF-*α* (*t* (10) = 6.273, *p* < 0.001) and IL-1*β* (*t* (10) = 8.891, *p* < 0.001). Furthermore, the ethanol-induced activations of the TNF-*α* (*t* (10) = 7.354, *p* < 0.001), IL-1*β* (*t* (10) = 2.835, *p* < 0.05), and IL-6 (*t* (10) = 6.324, *p* < 0.001) were further increased in the SENP6-siRNA-transfected cells. These results strongly support that SENP6 contributes to the regulatory mechanism of the ethanol-induced activation of NF-*κ*B and the release of inflammatory cytokines in the microglia.

## 4. Discussion

Microglia-mediated neuroinflammation is a hallmark feature of most neurodegenerative diseases [38, 39]. Numerous studies have observed microglia activation and proinflammatory cytokine release after ethanol exposure [16, 40–42]. Furthermore, studies have indicated that blockade of TLR4 activation inhibits the microglia-induced increases of Iba-1 immunoreactivity and proinflammatory cytokines in the EtOH-treated microglia [24–27], which supports a key role of TLR4 in regulating ethanol-induced microglial activation and neuroinflammation [32, 43]. The present study found that ethanol exposure increased Iba-1^+^ and ED-1^+^ cells in the CA1 CA2/3 and DG regions of the hippocampus, decreased IkB-*α* expression, and increased p-NF-*κ*B-p65, as well as TNF-*α* and IL-1*β* levels in the hippocampus. These data suggest that chronic alcohol exposure drives the microglia into a phagocytic activated proinflammatory state.

It is noticed that the present study demonstrated that TLR4 expression only markedly increased in the CA2/3 region of the WT ethanol-treated animals but not in the CA1 and DG subfields. However, the difference of TLR4 expression in the CA1 region between the WT and WT+EtOH groups was marginally significant (*p* = 0.052). The results indicated that ethanol induced more TLR4 expression in the CA regions of the hippocampus compared with the DG subfield. Meanwhile, the percentage of phagocytic activated microglia in the CA regions (CA1 and CA3) was significantly increased in the ethanol-exposed groups compared to the controls in the hippocampus of the developing brain [44], indicating that the CA regions of the hippocampus in developing rats are more susceptible to ethanol stimulus. In addition, the TLR4 protein expression was almost undetectable in the KO group and was expected to be low in the KO+EtOH group. The findings confirmed the fine modification of TLR4 protein levels in TLR4 knockout microglial cells. Moreover, combination with knockdown of TLR4 prevented ethanol-induced increases in immunoreactivity against Iba-1 and ED-1, as well as p-NF-*κ*B-p65, TNF-*α*, and IL-1*β* levels. Our results demonstrate that TLR4 plays a pivotal role in the ethanol-induced activation of microglia and release of proinflammatory cytokines which is consistent with the previous studies [24–27].

Recently, SENP6 was reported to be involved in the regulation of NF-*κ*B activation [32, 33], which acts as a pivotal nuclear transcription factor in the TLR4-inflammatory signal pathway [28]. SENP6 can dampen the IKK activation via the de-SUMOylation of NEMO [32]. The overexpression of SENP6 significantly decreased the LPS-induced release of proinflammatory cytokines, while the depletion of SENP6 increased these cytokines [33]. In the present study, the SENP6 level was significantly decreased in the hippocampus after 21 days of binge drinking alcohol in in vivo experiment, while knockdown of TLR4 significantly inhibited this process. Moreover, in in vitro study, ethanol dose dependently decreased the SENP6 level at the higher dose of 50 mM and 100 mM compared with the no-alcohol control group. These data suggest that ethanol may regulate the SENP6 expression by TLR4 activation. Generally, there are two ways to regulate protein expression level. One way is by protein degradation via various protein modifications, e.g., phosphorylation, SUMOylation, acetylation, and ubiquitination [45–47]. The other way is by the translation control point through miRNAs. One former study found that microRNA-669n (miR-669n) regulated the LPS- (the specific activator for TLR4) induced SENP6 protein expression at translational level [33]. Perhaps, this molecular pathway is also involved in ethanol/TLR4 signaling-controlled SENP6 loss. Future studies may be applied to examine whether miR-669n is modulated by ethanol for improving our understanding of the mechanism of SENP6 loss by ethanol. Importantly, the overexpression of SENP6 significantly dampened the ethanol-induced NF-*κ*B activation and increased release of the proinflammatory cytokines TNF-*α* and IL-1*β* in microglial cells. And the ethanol-induced activation of the p-NF-*κ*B-p65 and neuroinflammation were further markedly enhanced in the SENP6-siRNA-transfected cells. These data first highlighted that the SENP6 plays an essential role in dampening ethanol-induced NF-*κ*B activation and neuroinflammation in the microglia. Future in vivo studies should be performed in both adolescent and adult rat brains to improve the completeness of our understanding of the regulatory role of SENP6 in dampening ethanol-induced NF-*κ*B activation and neuroinflammation.

## 5. Conclusions

In summary, the current study firstly demonstrates that 21 days of alcohol exposure during adolescence induces a phagocytic activated proinflammatory state of microglia through TLR4 activation. More importantly, we confirmed that SENP6 plays an essential role in dampening ethanol-induced NF-*κ*B activation and neuroinflammation in the microglia. The findings may provide important clues regarding SENP6 being involved in neuroinflammation induced by alcohol of the adolescent hippocampus. Further research should be made to investigate the other posttranslational modifications of TLR4 signaling-related factors, other than NEMO, involved in this process.

## Data Availability

All data used to support the finding of this study are available from the corresponding author upon request.

## Supplementary Materials

Supplementary Materials Supplementary Table 1: blood ethanol concentration data. The table showed the blood ethanol concentrations collected weekly (1st week, 2nd week, and 3rd week) at two time points (2 h after the onset of light and dark) per day in the WT+EtOH and KO+EtOH groups. The blood samples were obtained from the tail vein, and the values were averaged in each group (*n* = 10) at any time point. Supplementary Table 2: microglia purity data. The table showed the purity data of the cultured microglia. The cell culture was stained by immunofluorescence using anti-Iba-1 and DAPI for purity identification. The nuclei in the DAPI staining appeared blue, and Iba-1-positive cells appeared red. The merging of blue and red was considered positive cells (microglia). All counting was performed by researchers who were blinded to the groups. Cells in seven random fields (200x magnification) were quantified and averaged. The positive rate (%) = number of positive cells/total number of nuclei × 100%.
