# Supplementary material for: Ethanol Exposure Induces Microglia Activation and Neuroinflammation through TLR4 Activation and SENP6 Modulation in the Adolescent Rat Hippocampus
Source: Neural Plast. 2019 Nov 12;2019:1648736. doi: 10.1155/2019/1648736 (PMC6874951; doi:10.1155/2019/1648736)
Supplement: Supplementary Materials — Supplementary Table 1: blood ethanol concentration data. The table showed the blood ethanol concentrations collected weekly (1st week, 2nd week, and 3rd week) at two time points (2 h after the onset of light and dark) per day in the WT+EtOH and KO+EtOH groups. The blood samples were obtained from the tail vein, and the values were averaged in each group (n = 10) at any time point. Supplementary Table 2: microglia purity data. The table showed the purity data of the cultured microglia. The cell culture was stained by immunofluorescence using anti-Iba-1 and DAPI for purity identification. The nuclei in the DAPI staining appeared blue, and Iba-1-positive cells appeared red. The merging of blue and red was considered positive cells (microglia). All counting was performed by researchers who were blinded to the groups. Cells in seven random fields (200x magnification) were quantified and averaged. The positive rate (%) = number of positive cells/total number of nuclei × 100%. [file 1648736.f1.pdf]

Supplementary Table 1 Blood ethanol concentrations data

Group		N	1st BEC (mg/dl)	2nd BEC (mg/dl)	3rd BEC (mg/dl)
WT+EtOH	2h after light onset	10	33 ± 3.1	36 ± 3.4	33 ± 3.6
	2h after light offset	10	98 ± 5.5	108 ± 8.9	107 ± 8.9
KO+EtOH	2h after light onset	10	34 ± 3.3	35 ± 2.7	33 ± 3.5
	2h after light offset	10	97 ± 7.5	101 ± 8.6	97 ± 7.0

Supplementary Table 1. Blood ethanol concentrations data
The table showed the blood ethanol concentrations collected weekly (1st week, 2nd week, 3rd week) at two-time points (2 h after the onset of light and dark) per day in the WT+EtOH group and KO+EtOH group. The blood were obtained from the tail vein and the values were averaged in each group (n=10) at any time point.

Supplementary Table 2 Microglia purity data

Field (×200)	Number of Positive cells (cells/field)	Total Number of Nuclei (cells/field)	Positive rate (%)
1	308	325	94.77
2	311	322	96.58
3	288	303	95.05
4	299	316	94.62
5	317	331	95.77
6	307	324	94.75
7	301	315	95.56

Supplementary Table 2. Microglia purity data
The table showed the purity data of the cultured microglia. The cell culture was stained by immunofluorescence using anti-Iba-1 and DAPI for purity identification. The nuclei in the DAPI staining appeared blue, and Iba-1-positive cells appeared red. The merging of blue and red were considered positive cells (microglia). All counting was performed by researchers who were blinded to the groups. Cells in seven random fields (200× magnification) were quantified and averaged. The positive rate (%) = number of positive cells/total number of nuclei×100%.
